# Perceptions of managerial staff on the patient safety culture at a tertiary hospital in South Africa

**DOI:** 10.1080/17482631.2022.2066252

**Published:** 2022-04-21

**Authors:** Veena Abraham, Johanna C Meyer, Brian Godman, Elvera Helberg

**Affiliations:** a Division of Public Health Pharmacy and Management, School of Pharmacy, Sefako Makgatho Health Science University, South Africa; b Strathclyde Institute of Pharmacy and Biomedical Sciences, Strathclyde University, Glasgow, UK; c Centre of Medical and Bio-Allied Health Sciences Research, Ajman University, Ajman, UAE

**Keywords:** Patient safety culture, South Africa, quality, hospitals, healthcare professionals, managers

## Abstract

**Background:**

Hospital managers are best suited and located to enhance patient safety culture within their institutions.

**Aim:**

This study sought to provide insight on the perceptions of 10 managerial staff regarding the patient safety culture at a tertiary academic hospital in South Africa.

**Method:**

In-depth interviews were conducted with strategic and operational managers within high-risk units in order to determine their perceptions regarding patient safety culture.

**Findings:**

Participants described diverse aspects of the hospital’s patient safety culture including an overview of patient safety, implementation of patient safety initiatives, challenges to patient safety, current management of patient safety issues, as well as ways to improve the patient safety culture of the hospital.

**Conclusion:**

The findings highlighted a number of areas to improve on to advance patient safety within the South African context. These include improving basic services, strengthening the infrastructure, improving staff attitudes and implementation of patient safety initiatives. Further research and development of quality improvement plans are essential to enhance patient safety.

## 1. Introduction

Managerial and clinical staff perceptions of patient safety vary significantly, often differing in terms of prioritizing interventions that could improve patient care and safety, as well as their perceived roles in establishing and maintaining patient safety (Parand et al., 2011; Quenon et al., 2020). Hospital managers, by failing to recognize their own role in supporting patient safety, may actually hinder patient safety and error reporting. This includes concerns with the effectiveness of handover of patients between staff members at crucial times (Richter et al., 2016). It has also been demonstrated that even with management input and support, errors remain underreported (Richter et al., 2015).

In general, patient safety is defined as *“the avoidance and prevention of patient injuries or adverse events resulting from the processes of health care delivery”* (AHRQ, 2021) while the term culture commonly refers to the *“values, attitudes, norms, beliefs, practices, policies, and behaviours of personnel”* (Pronovost & Sexton, 2005). Furthermore it has also been suggested that the patient safety culture of an institution refers to *“the way we do things around here”*, and that culture is a confined singularity, referring in particular to a work unit (Pronovost & Sexton, 2005). Combining these concepts, patient safety culture has been defined as “*a set of values attitudes, perceptions, beliefs, and behaviours that support the safe conduct of individuals’ activities in health organizations*” (Khoshakhlagh et al., 2019). Other authors have also emphasized that the physical environment within an institution plays a role in patient safety as well (Joseph et al., 2012).

To improve patient safety, Richter et al. (2015) suggested that managers provide interventions such as patient safety subcommittees as well as incentivize good safety performances among staff. Parand et al. (2011) indicated the need for managers to approach error reporting as a form of organizational learning, to improve upon safety efforts and to motivate staff to report errors. We see exactly the same situation when it comes to pharmacovigilance activities in hospitals in South Africa and wider, and the need to instigate a culture of reporting and learning (Katusiime et al., 2015; Shamim et al., 2016; Terblanche et al., 2017). Existing organizational patient safety cultures can act as a double edged sword though. In some instances, promoting excellent care whilst in others concealing poor patient safety practices (Dixon-Woods, 2010).

Hospital managers are ideally positioned to ensure organizational policies and systems do enhance a patient safety culture within institutions. They can play an important role in maintaining the quality of care to patients, with managerial attitudes playing an important role in the development of a mature safety culture within hospitals (Pierre, 2013). The patient safety culture of an organization is usually established by the senior management and executives within hospitals, with managers potentially incentivized to promote a just and positive patient safety culture within their institutions.

This is illustrated in the USA where from 2008, Medicare has stopped paying hospitals for errors deemed preventable with hospitals becoming liable for any payments that arise from errors including additional procedures or extended hospital stays (Brooks, 2007). Similarly in South Africa, the National Department of Health (NDoH) has certain policies and guidelines that inform best practices across the spectrum of healthcare providers. These include the NDoH (2007) recommending interventions focussed on improving care by healthcare professionals, such as continuing professional development, the use of structured encounter forms such as prenatal care forms, and providing feedback to clinicians regarding their performance as means to improve upon quality of care.

The NDoH also introduced National Core Standards in 2011, as well as the National Policy in 2015 to better manage patient safety incidents within the public healthcare sector to further improve patient care in South Africa 2007,2015,2011 (. Healthcare institutions in South Africa are also subject to annual quality audits by the NDoH, the results of which determine how much funding will be allocated to the institution. Consequently, there are financial implications and incentives for healthcare institutions in South Africa to successfully implement patient safety initiatives. Other initiatives to generally enhance the quality of and efficiency of care in hospitals including establishing and strengthening the role for Pharmaceutical and Therapeutics Committees (PTCs) within hospitals to improve the monitoring of medicines and reduce errors (Mashaba et al., 2019; Matlala et al., 2020; Meyer et al., 2017).

This is important when the provision of care to patients is unsafe, and in worst case scenarios, causes them harm. This can lead to costly litigation against healthcare providers or the hospital as a whole (Sohn, 2013). In the Gauteng Province of South Africa, more than 20,000 patients have been harmed in hospitals over a period of two and a half years from January 2016 to June 2018 (TIMESLIVE 2018). In addition, in both 2019 and 2020, the number of serious adverse events in Gauteng hospitals was over four thousand (Molelekwa, 2021). Often these cases are highly publicized and negatively affect the public’s perceptions regarding particular hospitals. Managerial support and engagement can help address such attitudes since their subsequent activities have been associated with positive quality outcomes (Parand et al., 2014). One of the ways to improve healthcare quality is determining managerial perceptions regarding the current patient safety culture within their hospitals and the factors that influence this (Nordin et al., 2013).

In view of this, the aim of this study was to establish the perceptions of managerial staff regarding patient safety culture at a leading tertiary public hospital in South Africa. We chose a public tertiary hospital as a starting point because we believed that concerns ascertained in public tertiary hospitals would be echoed in regional and district public hospitals. In addition, the public healthcare system takes care of 80% of the population within South Africa, with recent strides to introduce universal healthcare in South Africa (Meyer et al., 2017). The findings can subsequently be used to guide future activities and suggestions within public hospitals in South Africa and wider.

## 2. Method

The study was conducted utilizing a qualitative descriptive research design. In-depth interviews were conducted with strategic and operational managers within high-risk units in order to determine their perceptions regarding patient safety culture.

### 2.1 Setting and participants

The study was conducted at one of the ten tertiary hospitals in South Africa. Purposive sampling was used, according to the hospital’s organizational structure, to recruit participants at a managerial level. According to the hospital’s management structure, the Director of Clinical services is in charge of all clinical staff within the Departments of Nursing, Allied Services, Medical Services, Mother and Child Health and Quality Assurance. The Director of Clinical services as well as managers and operational managers from these departments were purposively recruited to reflect the diverse views relevant to this hospital and its context. Participation was dependent on willingness to participate and availability. Data saturation was reached after interviews had been conducted with 10 managers, when no new themes emerged from the data and no further coding was feasible, in line with established guidance (Fusch & Ness, 2015). Seven of the 10 participants were female, including seven Africans, two Caucasian and one coloured or “mixed-race”. All three male participants were Africans. Further participant demographics are outlined in Table I.

**Table I.** Participant demographics

Job title	Gender	Race	Professional qualification/role
Clinical Executive: Medical and Mother and Child	Female	Caucasian	Medical practitioner
Deputy Director of Allied Health Services	Female	Caucasian	Allied health professional (Dietician)
Head of Department of Paediatrics and Child Health	Female	African	Medical practitioner
Chief Specialist: Department of Family Medicine and Primary Health Care	Male	African	Medical practitioner
Deputy Director of Nursing	Female	Coloured (Mixed-race)	Nurse
Nursing Manager	Female	African	Nurse
Head of Internal Medicine	Female	African	Medical practitioner
Clinical Pharmacist and Manager	Male	African	Allied health professional (Pharmacist)
Quality Assurance Manager	Female	African	Nurse
Director of Clinical Services	Male	African	Medical practitioner

### 2.2 Data collection

An interview guide, allowing for flexibility of follow-up questions based on participants’ responses, was used to conduct semi-structured, in-depth interviews. The interview guide used in the study was developed by the first author for two purposes: i) as part of this initial qualitative exploration of managerial perceptions on patient safety; and ii) to inform the development of a self-administered questionnaire that was to be used as part of a follow-up survey on patient safety with frontline clinical staff. The interview guide was reviewed by two experts in the field of patient safety prior to use. Though the interview guide was not formally piloted, prompts during the first interview led to additional questions being added. The final version of the interview guide is available in Annexure A (Supplementary Material). The first author conducted all the interviews in English, the official language of communication in the workplace, with an independent observer present. Interviews were conducted in a private room at the participant’s convenience, typically in their own offices. All interviews were audio recorded and 30 to 45 minutes in duration.

In addition, participants were provided with a 5-point rating scale to evaluate the institutions’ current management of patient safety issues (Deutsch et al., 2015; Sabblah et al., 2017). The scale ranged from 0 to 4, with 0 indicating not at all effectively, followed by 1 indicating somewhat effectively, 2 quite effectively, 3 very effectively and 4 indicating extremely effectively. Using this scale assisted with gauging the overall managerial perceptions of patient safety at the institution and provided a baseline to compare to, in a follow-up survey with frontline clinicians.

### 2.3 Data Analysis

Audio recordings of the interviews were transcribed verbatim throughout the data collection period. Any information that could identify participants was removed from the transcript and each respondent was assigned a unique number as an identifier. The authors subsequently read and re-read each interview to ensure identification of any aspects that would need additional probing in subsequent interviews. Transcripts were subsequently imported into NVivo® 10 software (QSR international, 2010) for coding and a thematic analysis of the data according to established frameworks (Braun & Clarke, 2006; Richards, 2020; Vaismoradi et al., 2016, 2013). Line-by-line open coding was used by the first two authors to independently code the first interview. The first author is a scientist and lecturer in pharmacy and was a Master’s student at the time of the study, while the second author is a pharmacist and Professor with extensive experience in both private and public health services research in South Africa and abroad, utilizing both quantitative and qualitative research methods.

The codes were compared, discussed and modified by the two coders to develop an initial codebook for further coding. As coding progressed, continuous discussions took place between the two coders, with existing codes being modified and new codes generated. Codes were grouped into initial themes until the point of data saturation was reached and no further coding was feasible. Themes were then corroborated to conclude on the final main themes and sub-themes. The coded output was continuously reviewed and discussed by all authors.

All grammatical and typographical errors were corrected to enhance the readability of any quotes used to illustrate themes and sub-themes. The use of ellipsis in quotes indicates text omitted from the original quote (without changing the meaning of the quote), while square brackets were used to further explain the quote context. To maintain confidentiality, only professional designation was subsequently used to identify participant quotes.

### 2.4 Ethical considerations

Prior to conducting the study, ethical clearance was obtained from the Sefako Makgatho University Research Ethics Committee (SMUREC/H/28/2015:PG). Permission to conduct the study was granted by the Chief Executive Officer and the Clinical Director of the hospital. All participants were given written information about the study and written informed consent was obtained upon agreement to participate prior to the interviews. Participation was voluntary and confidentiality was assured.

## 3. Results

**Figure 1.**
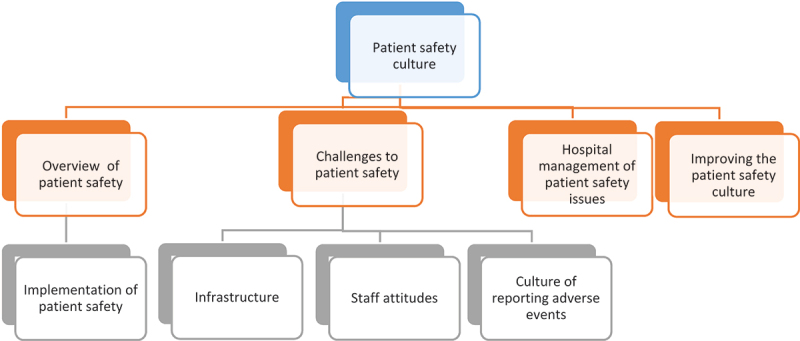
An overview of the patient safety themes and sub-themes.

Four main themes with underpinning sub-themes emerged from the interviews with ten managers within the Clinical Services Directorate. A diagrammatic representation of the identified themes is presented in Figure 1.

### 3.1 Overview of patient safety

Participants elaborated on their understanding of the term “patient safety culture”. They were also asked on how they implement patient safety within their units. Participants unanimously perceived patient safety culture as protecting patients from any form of harm that may arise as a result of the care given, and that it stems from the ideals and work ethics of various staff members within the hospital hierarchy. The protection from harm encompasses both physical harm and security for patients, the quality of care provided to patients and the attitudes of staff towards patients. Participants also understood patient safety culture as adherence to the standards set by the NDoH.

A common theme that emerged during this stage of the interviews was that for a positive culture to exist, all staff and the hospital as a whole have to be proactive about patient safety, rather than reactive:

… when an event takes place that’s related to patient safety, it’s always reactive, across the board, whatever kind of patient safety, it’s reactive, write reports and people perceive it as punitive, that something is going to happen that’s going to be negative. (Respondent 04, medical practitioner)

What we are doing is not well coordinated, and we need, on a regular basis, just to have talks on patient safety. We need it. Both to the doctors and to the nurses … as we talk about patient safety, we want to prevent it, we don’t want to act after the fact. (Respondent 07, medical practitioner)

#### 3.1.1 Implementation of patient safety

Most participants highlighted physical security measures that are put in place to aid patient safety. Access to high risk areas including paediatric wards and neonatal intensive care units is controlled via security gates, and security guards are on duty at all times.

Policies and procedures regarding patient safety appear well established throughout the hospital, however participants indicated that implementation of these are sometimes lacking, often resulting in poor patient safety awareness filtering down to members of staff at the hospital.

In each ward there is a file with all the standard operating procedures, we also have the issue of the National Core Standards … if it’s just an operational plan you just put it in the cupboard and you go on anyway with what you are doing, it won’t assist. (Respondent 01, medical practitioner)

Participants stressed the importance of hospital staff compliance with patient safety efforts as patient safety falls under the National Core Standards as laid out by the NDoH and, as such, patient safety is one of the priority areas for improvement for the NDoH in South Africa. In certain units such as paediatrics, internal medicine and family medicine, there are weekly mortality and morbidity meetings that take place to address potential issues that could have caused errors or adverse events and how to improve on the situation. However, this is not universal:

… it’s only very few departments that have mortality and morbidity meetings in this hospital. Our department documents adverse events, which ones were unavoidable. We sort of review them in detail and we do that on a weekly basis. (Respondent 04, medical practitioner)

Some participants mentioned that staff training and workshops on patient safety do take place within the hospital. The presence of multidisciplinary committees such as the adverse event committee, and quality assurance committee, was seen to be an important way of implementing the patient safety culture of the hospital, as reiterated by participants:

We have what we call a serious adverse event committee that focusses mostly on very serious adverse events, in other words, deaths … it will be investigated and the committee will see how we can remedy the situation … and to prevent the same thing from happening again. I get monthly reports from the nurses [about other adverse events], why and what happened, what remedial action did they take with regards to that. (Respondent 01, medical practitioner)

### 3.2 Challenges to patient safety

Several of the participants gave the impression that the current patient safety culture of the hospital was sub-optimal in certain aspects. A number of factors that impede the establishment of a positive patient culture at the hospital were identified by them. These included infrastructure, staff attitudes, lack of patient safety awareness and poor adverse event reporting.

#### 3.2.1 Infrastructure

Overwhelmingly, participants described infrastructural factors as important to patient safety. Maintenance issues such as lack of air-conditioning, broken doors, and lack of security gates in certain wards, all had a negative impact on the patient safety culture within the hospital. Leaking water pipes and a shortage of functional generators were also highlighted. One participant mentioned that at one point there was no water in the hospital, which lead to an infection outbreak as staff could not wash their hands before attending to patients. Maintenance work undertaken by contract workers was seen to be of sub-optimal quality and not well maintained:

Ok I think the primary issues here would be like the facility itself, safety of the facility itself, and the environment ….the air conditioners do not work so the wards are very hot … Sometimes we do have leakages … the pipes are old and rusted and leaking, so the infrastructure itself is a primary issue … we need to have within the hospital structure, an area for isolation of infected patients, we don’t have adequate isolation rooms with proper ventilation. (Respondent 06, nurse)

According to our reports [the lack of water] is the cause of the outbreak that we had, we would say wash your hands before you touch a baby, but how do you wash your hands in winter with cold water? At first there was no water, then there was cold water but no hot water, so how do you balance the two? (Respondent 08, allied health professional)

Participants discussed the importance of maintaining the cleanliness of the hospital, as well as the availability of clean linen. Participants highlighted that a lack of resources significantly contributed to infrastructure issues and staff shortages. As the study site is a tertiary academic hospital, many participants commented that the size of the hospital itself hinders the delivery of safe patient care.

And this hospital is a bit big as well, I’m not sure if it will ever be possible to run a very safe hospital if it stays this big. Maybe if it’s a bit smaller, I think it’s easier to control. There’s always a lot of patients, so many. So it would help if there’s a referral hospital somewhere or if the patients can just be down referred somewhere. (Respondent 02, Allied Health Professional)

#### 3.2.2 Staff attitudes

All participants indicated that staff attitudes towards their work and patients affect patient safety. Participants claimed that clinical staff appear no longer to have a comprehensive culture of caring for patients, and can be rude and dismissive of patients at times. Participants alluded to the fact that most complaints filed by patients or their family members stem from the attitudes of the staff towards the patients or their care.

Staff attitudes were thought to be linked to staff members being overworked due to staff shortages as well as to the high number of patients being admitted to the hospital on a daily basis. Participants alluded that staff being overworked often led to staff burnout. This shortage of staff is especially true of specialized nurses. The private sector also appeals to nurses due to better pay and fewer patient numbers. Retention strategies included training nursing staff in management skills and patient safety; however, participants still lamented the high staff turnover rate. Participants unanimously agreed that of all the clinical staff involved with patients, nurses are the staff who spends the most time with patients. Consequently, they are a key group to improve any patient safety culture within hospitals.

Attitude also plays a role, but that also need to be linked to staff being burnt out, if you have adequate staffing, you are not likely to experience burnout, you actually can help the attitude, but if you are burnt out it is easy to take out frustrations and unfortunately that feeds to a weaker person, the patient, they can’t defend themselves, they are sick. (Respondent 08, allied health professional)

#### 3.2.3 Culture of reporting adverse events

Concerns were raised by participants that there is a lack of adverse event reporting at the hospital. When reporting does occur, the consensus from participants was that a culture of “blame and shame” pervades the lower levels of staff. Staff are reluctant to report incidents or adverse events as they perceive this to have a punitive response, which is a concern. Staff members feel that their mistakes are held against them and that they may lose their job over a serious error. The participants also suggested that a fear of litigation was another reason that reporting adverse events is scarce in this setting.

I think it’s across the board, you’ll find the nurse will not want to report it but may have to write it in the occurrence booklet, they have to report what happened at each shift. Whereas the doctors may be scared to say, “If I report it now, what’s going to happen? Am I going to face the law?” So it’s an issue of, people don’t look at it as a way of improving the quality. They think it’s going to be very punitive, like someone is going to hit them hard with … Write a report. And I think people are just scared they’ll be reprimanded, have a finger pointed at them. (Respondent 04, medical practitioner)

**Figure 2.**
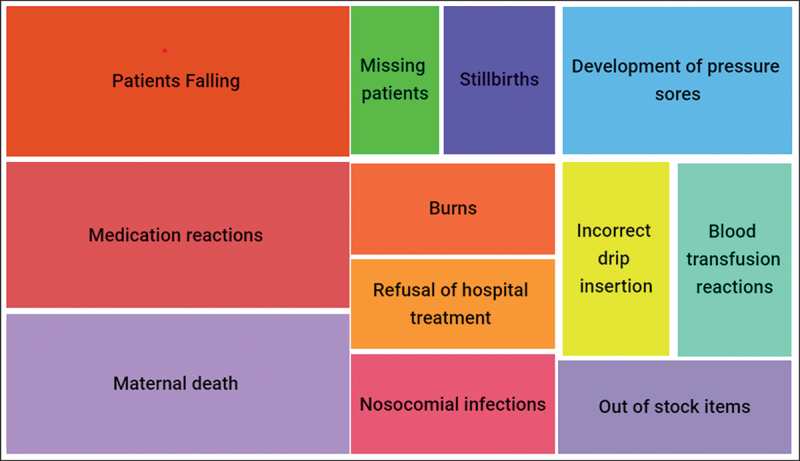
Common adverse events that occur in the hospital according to participants.

Participants agreed that adverse event reporting should be seen as a learning opportunity and that quality improvement plans could be developed if there was error reporting in the hospital. As a starting point, participants were asked to list common adverse events that took place within their units (Figure 2).

### 3.3 Hospital management of patient safety issues

Nine out of the ten participants rated the hospital at the level of quite effectively or below with respect to the hospital’s current management of patient safety issues, with only one participant giving the hospital a rating of 3, i.e., very effectively. When probed on the reasons why participants gave a particular rating, the responses varied.

I would rather under-rate ourselves than give ourselves a rating that I am not comfortable with because all the processes and systems that we are busy to develop, we will see it in the years to come. For now, we just try to put things into place. (Respondent 05, nurse)

The reasons for my rating, well organizational culture and patient safety are not things we talk about, there is no system to report to and highlight patient safety within the hospital and also adverse events are just not being reported by staff members. (Respondent 03, medical practitioner)

I would say at the moment from quality department, we are developing some quality improvement strategies to address those causes of serious adverse events and currently we are also doing awareness like presenting them to the people to be aware and encouraging the operational managers to draw up their standard operating plans towards that and then to monitor that as well. We also have the recording where we get the reports from each and every unit on issues such as the falls and then we present them to higher management and staff as well so that we can address them and have redress and quality improvement plans towards that. So we are trying, the hospital is trying. (Respondent 10, quality manager)

Participants agreed that there were currently no proactive measures in place to prevent patient safety issues from occurring. Some participants stated that reporting systems were not currently well established and patient safety policy implementation was not possible.

Most of the participants stated that they did not feel comfortable giving a high rating for the hospital because there were still many gaps in terms of patient safety such as infrastructural deficiencies and lack of resources.

Participants agreed that adverse events do occur and that many cases of litigation are currently pending against the hospital. Participants felt that much more awareness and work needs to be put in place before they would be willing to give the hospital a higher rating in terms of managing patient safety issues.

### 3.4 Improving the patient safety culture

The majority of participants indicated that to improve the patient safety culture, staff needed to be caring, empathetic and respectful to patients and one another. Participants recommended that staff treat patients as they would treat themselves or their relatives.

For me, I would think that if you just hold to this [value] of treating another person like you would like to be treated, I think we’ll go a long way … Values like empathy and integrity but it can be words, you must actually live those values otherwise it just remains words. (Respondent 01, medical practitioner)

Participants recommended that staff need to be committed to their patients and emphasized that the importance of increased patient safety awareness within the whole hospital. One participant suggested that exemplary patient safety efforts by staff should be acknowledged by hospital management and possibly incentivized to improve future care. The following statements reflected their values to improve patient safety culture:

To care for a patient as if they are your relatives. It has always been my philosophy for many years that I see all patients as my relative so I’ll give the relative the best, I won’t give my relative the worst. If we all adopt that, you’ll find that, if I see a person falling down, you help the person up. (Respondent 04, medical practitioner)

Overall, all participants summarized their interviews with the idea that patient care is the core business of the hospital and for all clinical and nursing staff, and as such each individual needs to adopt patient safety as a personal priority.

Patient care is my primary concern … I’m here to serve the patient, I’m not here for anything else. (Respondent 01, medical practitioner)

Why are you a healthcare professional if you are not serious about patient safety? Then it’s the wrong place for you. (Respondent 05, nurse)

## 4. Discussion

We believe this is the first study within a tertiary hospital in South Africa to explore patient safety issues as the Government and others strive to improve care within the public healthcare system. Encouragingly, the concepts put forward by participants regarding what patient safety culture entails, tie in well with the established definitions of patient safety culture (Lawati et al., 2018; Pronovost & Sexton, 2005). Regarding the implementation of a patient safety culture within the hospital, participants elaborated on physical security measures to protect patients as well as the presence of policies, procedures and standards including the National Core Standards (NDoH,^2011^), multidisciplinary committees and meetings, and patient safety workshops.

Participants highlighted the various challenges faced by the hospital in ensuring patient safety. These included the issues of currently weak infrastructure, negative staff attitudes, lack of patient safety guideline implementation and a culture of not always reporting adverse events. Physical security and the physical environment have been shown to be important for patient comfort, patient safety and for establishing a conducive working environment for staff (Rashid, 2007); and concerns need to be addressed to improve future patient care. World Health, Organization & World Alliance for Patient Safety Research Priority Setting Working Group (2008) points out that a consequence of poorly maintained infrastructure and equipment in hospitals, especially in developing countries, is a higher incidence of adverse events, which must be avoided in the future (World Health, Organization & World Alliance for Patient Safety Research Priority Setting Working Group, 2008). The importance of a proper infrastructure for patient safety was also pointed out by the researchers (Urlich et al., 2004) who found that an optimal physical environment reduces staff stress and fatigue, improves patient safety and improves the overall quality of healthcare provided. Hrickiewicz (2015) also underscored the vital contribution that a hospital’s building infrastructure including technology and engineering infrastructures provides to the provision of quality care and patient safety. Poor infrastructure including the physical environment and human resources affects staff performance leading to human error, which in turn leads to adverse events that are essentially preventable. In terms of infrastructure, participants reported that encouragingly the hospital does have measures in place to ensure physical security of staff and patients alike, which is encouraging. The presence of security guards, CCTV cameras, and restricted access to certain areas, bears testimony to these efforts. However, there are an appreciable number of infrastructural issues in this hospital that hinder the establishment of a positive patient safety culture. These include the poor condition of the hospital building, lack of generators and poor water supply. These are similar to the findings in two hospitals in East Africa where the physical environment as well as a lack of equipment and supplies were seen to negatively affect healthcare workers’ ability to provide quality and safe care to patients (Aveling et al., 2015). In addition, participants lamented the lack of proper ventilation and inadequate isolation rooms, both of which have been identified as effective measures preventing the spread of hospital acquired infections, which can appreciably impact on morbidity, mortality and costs (Manoukian et al., 2018; Murni et al., 2015; Perovic et al., 2020; Urlich et al., 2004). Proper hand-hygiene is a critical standard for infection control in all hospitals (Allegranzi & Pittet, 2009; Ara et al., 2019), and the lack of water supply was hypothesized by one participant to be the cause of an infection outbreak in the wards. This needs to be addressed and is even more important with the recent COVID-19 pandemic, especially given concerns with high levels of antimicrobial prescribing in hospitals of patients with COVID-19 despite limited bacterial and fungal infections (Langford et al., 2021, 2020). High prescribing rates will accelerate antimicrobial resistance unless addressed (Godman et al., 2021; Hsu, 2020).

Participants in this study also discussed how staff are being overworked due to low staff numbers as well as increasing patient numbers, with current high turnover rates adversely affecting workloads, leading to burnout, stress and dissatisfied staff. Poor retention of staff, particular nurses, was highlighted as an area of concern by many of our respondents, with high turnover rates also seen in other studies (Stone et al., 2008). Aveling et al. (2015) also found similar results in East Africa, with Urlich et al. (2004) acknowledging that high clinician turnover rates result in overworked staff, burnout and potentially adverse events, with Carayon and Gurses (2008) pointing out that high patient numbers per healthcare professionals can have a negative impact on subsequent provision of care and patient outcomes. When this occurs, patients in our study often complained via the Quality Department of the hospital and if no redress could be reached within internal processes litigation ensued. Complaints from patients or their families often relate to the attitudes of staff towards them including not caring as well as lacking empathy and lack professionalism, similar to other studies (Kroening et al., 2015; Van De Ven, 2014). Galloway (2011) describes the concept of “dignity in care” which encompasses promoting dignity and sensitivity into clinical practice to provide safer care. This concept encompasses healthcare professionals affording respect for patients as they would expect for themselves or for their relatives, with attitudes and behaviours of staff significantly affecting dignity in care. Our recommendation based on the published literature and feedback from participants is for the hospital to invest in both infrastructure and human resources to provide safe and high quality care in line with published advice (Pronovost et al., 2008). In addition, encourage staff members to put themselves in the shoes of the patient and to treat them as they would treat their own relatives. We will be following this up in future research projects.

Participants all felt that current documentation and guidelines relating to patient safety, including the National Core Standards (NDoH, 2011), and the National Policy to Manage Patient Safety Incidents (NDoH, 2015), were well known and established within the hospital but that full implementation of these guidelines was lacking. This is a concern as organizations that practice care based on best practices such as national guidelines and protocols usually present a positive patient safety culture (Sammer et al., 2010). Principles acquired from safety awareness campaigns and workshops in the hospital were also not being implemented by staff, which is also a concern and should be investigated further.

Overall, it is believed that quality improvement interventions that align closely to the current clinical practices are easier to implement, with multifaceted interventions typically resulting in a greater change in the subsequent quality of care (Ranji & Shojania, 2008). In view of this, we suggest this hospital focuses initially on patient safety practices that are easier to implement and that are evidence-based. This includes implementing strategies to address the current culture that results in poor levels of reporting of adverse events. We have seen similar poor levels of reporting of adverse events from medicines across South Africa prior to instigating initiatives to address this (Dheda et al., 2016; Joubert & Naidoo, 2016; Terblanche et al., 2017, 2018). The importance of reporting adverse events was emphasized by participants who recognized it as a form of organizational learning, where quality improvement could be a result of improved reporting systems. However, this may be hampered by a culture of “blame and shame” that currently pervades among staff in the hospital, and that reporting adverse events held a punitive connotation for staff mirroring the findings in other studies (Heard et al., 2012). The current response to adverse events in the hospital was seen as predominantly reactive in nature. However encouragingly, the general consensus from participants was that the hospital was trying hard to improve the situation and improve patient care; however, many gaps still remain suggesting key areas for improvement.

This pervasive culture of fear regarding reporting adverse events could be eliminated by an organizational culture that places emphasis on safety and not on blame (Hughes, 2008), and that patient safety issues is often the result of system-wide issues and not due to an individual’s shortcomings (Woodward et al., 2009). Reporting all errors (including near-misses) that occur has the prospective benefit of strengthening the care process and improving the quality of care provided to patients (Hughes, 2008), which should be borne in mind going forward Participants also lamented the lack of feedback from reported incidents. However, this is changing with participants in certain units holding weekly mortality and morbidity meetings, during which incident reports or adverse events are discussed as a means to learn from the error and improve on service delivery. This mirrors the findings of Hor et al. (2010), who suggested mortality and morbidity meetings provide a platform for feedback to the units regarding the events that have taken place and discuss ways forward. Consequently, suggestions for this hospital going forward also include regular feedback meetings discussing adverse event reports and incidents to improve future care. Common adverse events in this hospital include patient falls, missing patients, misidentification of patients, adverse events due to drug treatment, poor infection control practices, pressure sores, unsafe blood products, maternal and new-born safety and mortality due to anaesthetic error, similar to the adverse events reported by De Vries et al. (2008). Overall, adverse incidents in developing countries are estimated to be of greater concern than those seen in developed countries due to a lack of resources and lack of implementation of positive patient safety practices (World Health Organization & World Alliance for Patient Safety Research Priority Setting Working Group, 2008), which need addressing going forward.

The values necessary for ensuring a positive patient safety culture include being caring, empathetic, and respectful of one another and patients. The overarching theme to improving the patient safety culture is that this is one of the main responsibilities of the hospital, and within this framework, each ward and each healthcare professional needs to adopt patient safety as a priority. Sammer et al. (2010) suggested that an ideal organizational patient safety culture comprises seven subcultures, namely (a) leadership; (b) teamwork; (c) evidence-based; (d) communication; (e) learning; (f) just; and (g) patient-centred. It was a concern in this hospital that despite most of the study participants being part of senior management, there was no mention of the role that leadership plays in cultivating a positive patient safety culture. If senior management is not investing appropriately in a patient safety culture, there is concern that this will not filter down. Communication as a foundation of a positive patient safety culture was also not discussed by the participants in our study, which is a concern for the future This is another core issue relating to patient safety and the lack of input on this topic from participants may be indicative of the current culture that exists, which needs to be urgently addressed. Improving the reporting of adverse events could facilitate the organization in learning from this experience and subsequently using the lessons learnt to develop future quality improvement plans promoting a culture of learning. A just culture is one where the reporting of adverse events is seen as a non-punitive process.

The ongoing COVID-19 pandemic has introduced an additional dimension to the already precarious healthcare system in South Africa. It has deepened existing inequalities by increasing mortality rates, affecting the mental health status of staff, increasing cases of gender-based violence, reducing physical activity (Mbunge, 2020). In addition, reducing the utilization of public health services such as HIV testing as well as clinics for patients with non-communicable diseases (NCDs) (Burger & Mchenga, 2021; Ogunleye et al., 2020; Pillay et al., 2021). Services for patients with NCDs including diabetes are increasingly important in South Africa and across Africa given rising prevalence rates, with management adversely affected by lockdown and other measures (Godman et al., 2020; Kluge et al., 2020). The pandemic has also increased daily costs for hospitalized patients (Edoka et al., 2021). The burden on the South African healthcare system is further confounded by the recent emergence of the Omicron variant, being more transmissible and partially resistant to existing vaccines (Torjesen, 2021). Healthcare resources and manpower are increasingly being diverted towards COVID-19 relief efforts, thus timely and effective efforts to improve patient safety in this setting are imperative, both during the pandemic and beyond.

## Limitations

The findings of this study should be interpreted in light of its limitations. Since this was a qualitative study, exploring the patient safety culture in a single institution, no inferences can be made from the findings beyond the study sample and therefore cannot be generalized to other institutions. Similarly, only managerial perceptions were recorded as part of this study, thus the results cannot be taken as representative of other staff members at the hospital. Another limitation was the refusal of some managers to participate in the study, although saturation of potential themes was reached with the included participants. However, despite these limitations, we believe the findings from this study provide valuable insights into the patient safety culture within public hospitals in South Africa, which need to be addressed going forward.

## Conclusion

Patient safety is a central pillar of any healthcare system. However, factors such as poor infrastructure, understaffing, and lack of access to basic equipment often preclude patient safety in these settings. A weak organizational patient safety culture coupled with disinterested leadership weakens any efforts to provide safe quality care to patients.

Participants in our study identified both strengths and gaps regarding the organizational patient safety culture. The importance of improving basic services (infrastructure, staff attitudes, training and implementation of safety initiatives) to advance patient safety illustrates that issues that affect patient safety reach far beyond hospital management and as such, require appropriate interventions.

Recommendations stemming from our research include investing in infrastructure and human resources, and implementing patient safety workshops/training sessions. However, further research and targeted quality improvement interventions are necessary to bring about tangible changes in the patient safety culture in this hospital.

## Supplementary Material

Supplemental Material
