# Supplementary material for: Perceptions of managerial staff on the patient safety culture at a tertiary hospital in South Africa
Source: Int J Qual Stud Health Well-being. 2022 Apr 21;17(1):2066252. doi: 10.1080/17482631.2022.2066252 (PMC9037162; doi:10.1080/17482631.2022.2066252)
Supplement: Supplemental Material [file ZQHW_A_2066252_SM9905.docx]

Supplementary Material: Interview guide

**Patient safety culture at a tertiary hospital in Gauteng, South Africa: Perceptions of nursing and clinical staff**

**DATE:** _______________

**RESPONDENT NO:** _____________

**POSITION IN HOSPITAL:** _____________________

Welcome the participant to the interview and thank him/her for their willingness to participate in the study. Explain why there is an independent observer present.

Elaborate on the aim of the study to the participant and obtain written consent from him/her to take part in the study

Explain that the interview will be audio recorded as part of data collection, but assure participant that information provided will remain confidential.

Reassure the participant to speak freely and honestly so as to get a clear and relevant perspective for the study

Pose the following questions to the participant:

1. **How would you define patient safety culture in your own words?**
2. **What has been your experience in terms of the patient safety culture of this hospital?**
3. **How do you implement this culture within your unit?**
4. **How important is patient safety to you?**
   1. **Follow up question: In your opinion; do you think everyone in this hospital understands the importance of patient safety? Why or why not?**
5. **What would you consider to be the primary issues relevant to patient safety?**
6. **What types of adverse events related to patient safety are you aware of that take place within this hospital?**

**How does this hospital respond to such events?**

1. **In your opinion, how effective would you say the hospital is in managing patient safety issues? 0 = not at all effectively**

**1 = somewhat effectively**

**2 = quite effectively**

**3 = very effectively**

**4 = extremely effectively**

**Probe: Please explain the reasons for choosing your answer**

1. **What would you say are some of the challenges (if any) facing the hospital in terms of patient safety?**
2. **What key values, if followed, would help this hospital to compete and thrive in terms of patient safety?**

The researcher will ensure that all aspects of the interview have been sufficiently covered. If more information is required, the interviewer will prompt the participant to elaborate further on his/her given answers.

Finally ask the participant whether there is anything they would like to add to what has already been said and if they feel there is anything important or relevant to the topic that has not been discussed.

Thank the participant for his/her participation in the study

**Close the interview**
